# BTK autoinhibition analyzed by high-throughput swaps of SH2 domains

**DOI:** 10.1073/pnas.2502688122

**Published:** 2025-10-10

**Authors:** Timothy J. Eisen, Sam Ghaffari-Kashani, Chien-Lun Hung, Jay T. Groves, John Kuriyan

**Affiliations:** ^a^Department of Chemistry, University of California, Berkeley, CA 94720; ^b^California Institute for Quantitative Biosciences, University of California, Berkeley, CA 94720; ^c^Department of Biochemistry, Vanderbilt University School of Medicine, Nashville, TN 37240; ^d^Department of Chemistry, Vanderbilt University, Nashville, TN 37240

**Keywords:** kinases, B-cells, SH2 domains, BTK, Src module

## Abstract

Bruton’s Tyrosine Kinase (BTK), a Tec-family tyrosine kinase, resembles the Src and Abl kinases in that an SH2-SH3 module regulates the activity of the kinase domain, principally through an inhibitory interaction between the SH3 and kinase domains. In Src-family kinases, phosphorylation of a C-terminal tail latches the SH2 domain onto the kinase domain, stabilizing the inhibitory conformation of the SH3 domain; in Abl, interaction between the kinase domain and a myristoyl group on the N-terminal segment provides a similar latching function. The structure of autoinhibited BTK resembles that of the Src and Abl kinases, but BTK lacks an obvious SH2-kinase latch. To assess the role of the SH2 domain in autoinhibition of BTK, we generated hundreds of chimeric BTK molecules in which the native SH2 domain is replaced by other SH2 domains. We measured the fitness of these chimeric proteins using a high-throughput assay in T and B cells. Surprisingly, many SH2 domains increased fitness when substituted into BTK. Analysis of one set of chimeric proteins indicates that the increase in fitness stems from the ability of the substituted SH2 domains to disrupt BTK autoinhibition while maintaining phosphotyrosine targeting. Thus, although BTK lacks a specialized latch, distributed interactions between the SH2 and kinase domains stabilize the autoinhibitory conformation of BTK. While phosphotyrosine recognition can be conferred on BTK by evolutionarily distant SH2 domains, autoinhibition requires specific interactions with the kinase domain that arose through evolutionary refinement of the regulatory mechanism, and is less easily mimicked by heterologous SH2 domains.

## Significance

Bruton’s Tyrosine Kinase (BTK) is an important target in cancer treatment, motivating studies into its mechanism. Like many other tyrosine kinases, BTK contains an SH2 domain that binds to phosphotyrosine residues. However, unlike kinases in the closely related Src and Abl families, BTK lacks an obvious intramolecular latch that stabilizes its autoinhibited state. Using high-throughput methods that enable measurement of the effects of hundreds of SH2-domain replacements, we find that the SH2 domain is crucial for stabilizing the autoinhibited state of BTK. Electrostatic interactions between the SH2 and kinase domains serve to stabilize the SH2 domain in an inhibitory conformation, suggesting that specialized latching mechanisms were a later evolutionary refinement.

The discovery of the phosphotyrosine-binding SH2 domain in 1986 by Pawson et al. (1) revolutionized our understanding of tyrosine kinase signaling. There are now over 59,000 cataloged SH2 domains across eukarya (2). The proliferation of SH2 domains in many different contexts, along with conservation of the phosphotyrosine-recognition site, highlights the broad utility of these modular units of phosphotyrosine recognition.

**Fig. 1.**
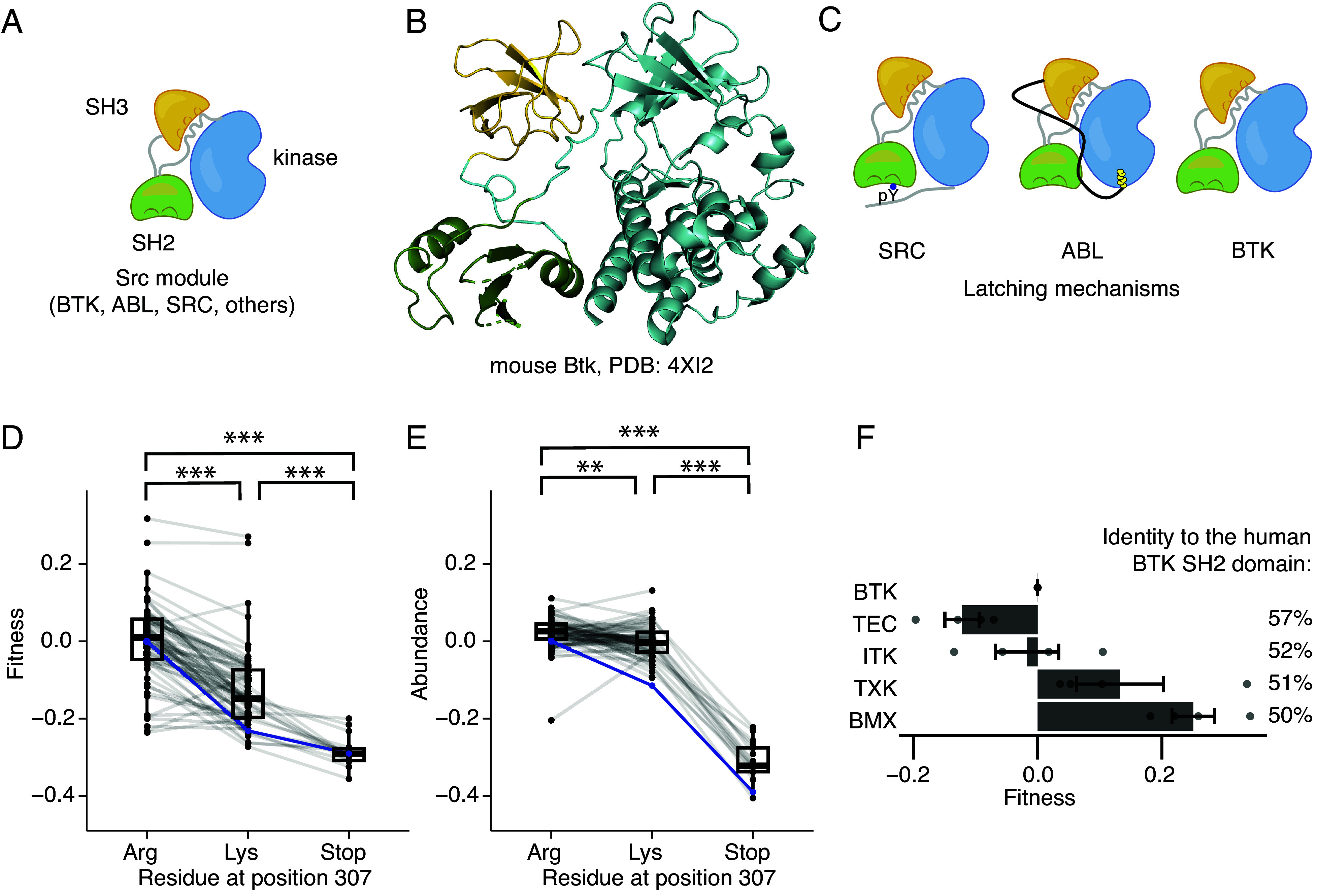
Phosphotyrosine binding of the BTK SH2 domain and chimeras. (*A*) A schematic diagram of the Src module. (*B*) A crystal structure of the Src module of mouse Btk (PDB: 4XI2; ref. 10), with colors as in (*A*). (*C*) Comparison between the autoinhibitory conformations of the Src modules in SRC, ABL, and BTK. (*D*) Fitness scores from mutation of Arg 307, the phosphotyrosine-interacting residue (shown in *SI Appendix*, Fig. S1*D*) measured using the Jurkat assay. SH2-domain sequences derived from the Tec kinases or ancestral-sequence reconstruction were mutated to replace the Arg residue at the equivalent position to 307 in human BTK with either lysine (62 sequences, including BTK) or a stop codon (17 sequences). The blue line is the human BTK SH2 sequence. A box-and-whiskers plot is overlaid with the line (median), box (first and third quartiles), and whiskers (1.5× the interquartile range). Asterisks denote ***P < 0.001 with a one-way ANOVA with Tukey’s multiple comparison tests. (*E*) Abundance measurements from the SH2-domain chimeras shown in Fig. 1*D*. Asterisks denote **P < 0.01 or ***P < 0.001 with a one-way ANOVA with Tukey’s multiple comparison tests. (*F*) Fitness scores from human BTK and the 4 BTK chimeras with human Tec-kinase SH2 domains are shown. The pairwise identity to the human BTK SH2 domain is shown at the *Right*. Error bars represent SEM and points are the individual replicate values.

In an important group of tyrosine kinases, including members of the Src, Abl, and Tec families, the SH2 domain is found within a “Src module” (Fig. 1 *A* and *B* and *SI Appendix*, Fig. S1*A*). The Src module is an ancient structural unit that consists of an SH3 domain, an SH2 domain, and a tyrosine kinase domain that arose before the emergence of the metazoan lineage (3). In Src-family kinases, the autoinhibited conformation of the Src module is stabilized by a latch, provided by a phosphorylated tyrosine residue in the C-terminal tail (residue 530 in human c-Src), that interacts with the SH2 domain (4, 5) (Fig. 1*C*). The latch anchors the SH2 domain next to the C-terminal lobe of the kinase and positions the SH2-kinase linker so that it can serve as a docking site for the SH3 domain. Interaction of the SH3 domain with the linker and the N-terminal lobe of the kinase stabilizes the inactive conformation of the kinase catalytic center (6, 7). The absence of the SH2 latch in the viral oncoprotein v-Src partly explains its constitutive activity (8). In the principal isoform of c-Abl (ABL1a), an N-terminal myristoyl group provides the latching function through an allosteric mechanism (9) (Fig. 1*C*). In the oncogenic BCR-ABL fusion protein, the myristoylation site is absent, contributing to uncontrolled kinase activity.

Bruton’s Tyrosine Kinase (BTK) is a member of the Tec family of tyrosine kinases, which contain a Src module preceded by a lipid-binding pleckstrin-homology (PH) domain fused to a Tec-homology (TH) domain (PH-TH module) and a long linker connecting the PH-TH module to the SH3 domain. BTK lacks an obvious SH2-kinase latch but the SH2 domain of BTK contributes to autoinhibition. A mutation in the SH2 domain (T316A) confers drug resistance in patients treated with the BTK inhibitor ibrutinib (11). The Src module of BTK adopts an assembled conformation that is very similar to that of c-Src and c-Abl, as shown by crystallography and cryoelectron microscopy (10, 12).

As in the Src and Abl kinases, a key autoinhibitory interface in BTK is between the SH3 domain, the SH2-kinase linker, and the kinase domain. The interface between the SH2 domain and the kinase domain is not tightly packed in BTK (10). An interaction between Arg 307 in the SH2 domain and Asp 656 in the C-terminal tail segment of the kinase domain contributes to BTK autoinhibition (13). The presence of a corresponding negatively charged residue in the C-terminal tails of the kinase domains in other Tec kinases suggests that these kinases may have evolved to use an aspartate or glutamate residue in place of the phosphotyrosine latch in the Src kinases. In BTK, the D656K mutation increases autophosphorylation of BTK and causes disassembly of the autoinhibited conformation (13). The importance of this single ion pair between Arg 307 and Asp 656 leads us to wonder whether there are other features of the SH2-kinase interface that support BTK autoinhibition.

In this study, we develop a high-throughput assay for BTK function that allows us to measure the relative fitness of hundreds of chimeric BTK molecules with different SH2 domains. The SH2 domains tested are derived from vertebrate Tec kinases, other human SH2-containing proteins, and SH2 domains generated using ancestral sequence reconstruction. As expected from the modular nature of SH2 domains, most SH2 domains are functional in BTK, with only 44 out of 249 chimeric BTK variants (17%) exhibiting strong loss of fitness. Even the most divergent SH2 domains tested, with as little as 25% sequence identity to the BTK SH2 domain, can maintain function when swapped into BTK. Surprisingly, 128 of the SH2 domains (51%) increase the fitness of BTK. For one set of SH2 domains, we trace the origin of these fitness increases to disruption of the SH2-kinase domain interface, demonstrating that this interface is important for BTK autoinhibition.

## Results

### Design of a Library of Chimeric BTK Proteins.

We constructed a library of BTK variants in which human BTK has its SH2 domain replaced with SH2 domains from BTK proteins from other species, from other Tec-kinases, or from other SH2-containing proteins (*SI Appendix*, Fig. S1*B*). After construction of the library of chimeric proteins, we tested the ability of these proteins to support signaling in lymphocytes, using an assay that we have described (14). The boundary of the SH2 domain was defined from the protein families [PFAM (2)] database of sequence alignments and corresponds to residues 281 to 362 in BTK. These alignments stem from a curated set of SH2 domains that are representative of the larger SH2 family. Although longer regions of homology can be detected between more closely related sequences, this aligned block of SH2 sequence facilitates searches for more distantly related domains.

We began by developing a computational pipeline to select SH2 domains to swap into BTK (*SI Appendix*, Fig. S1*C*). From the PFAM database, we used the five Tec kinase SH2 domain sequences to mine the PFAM database to select SH2 sequences from Tec kinases in different jawed vertebrates. The resulting set contained 83 Tec kinase SH2 sequences in which the most divergent sequence was that of the BMX SH2 domain from *Ursus maritimus* (the polar bear, with a pairwise identity of 46% with the human BTK SH2 sequence). We generated 114 additional SH2 sequences using ancestral-sequence reconstruction to interpolate between Tec-kinase SH2 sequences (15). The library also included SH2 sequences from proteins that are not Tec kinases (e.g., SRC, ABL1, SHIP-1, PTN11). Variant SH2 sequences, in which a critical phosphotyrosine-interacting arginine residue (Arg 307 in BTK, *SI Appendix*, Fig. S1*D*) was mutated to lysine, were also included. This mutation is expected to weaken phosphotyrosine binding substantially (16–18).

### Fitness and Abundance Measurements of Chimeric Proteins.

The fitness of each SH2–BTK chimera was determined using a cellular assay for BTK function (14) (*SI Appendix*, Fig. S1*E*). This assay relies on the ability of BTK or variants to induce expression of CD69, a surface-expressed glycoprotein commonly used as a marker for T and B cell activation (19). In one adaptation of the assay, heterologous expression of BTK variants in interleukin-2-inducible T-cell kinase (ITK)-deficient Jurkat T cells is used to measure their ability to support CD69 upregulation. The extent of CD69 upregulation, as determined by cell sorting, is defined as “fitness.” In the second adaptation of the assay, the fitness of BTK variants expressed in BTK-deficient Ramos B cells is determined in a similar fashion.

To measure fitness, lymphocytes expressing BTK or chimeric BTK variants were sorted based on their expression of CD69. High-throughput RNA sequencing (RNA-seq) was then used to measure the relative abundances of each chimera in the CD69-selected and input libraries, as described (14). RNA-seq is used instead of DNA sequencing because it detects more variants at a similar sequencing depth (14). We calculated fitness values for each chimeric protein *i* as follows:

$$\begin{array}{c} {\mathrm{Fitness}}_{i}=\log_{10}\left(\frac{{\mathrm{SortCount}}_{i}}{{\mathrm{InputCount}}_{i}}\right)\\ -\langle \log_{10}\left(\frac{{\mathrm{SortCount}}_{wild\;type}}{{\mathrm{InputCount}}_{wild\;type}}\right)\rangle . \end{array}$$

The values *Fitness_i_*, *SortCount_i_*, and *InputCount_i_* are the fitness score, RNA-seq read counts in the CD69-sorted library, and RNA-seq read counts in the input library for a particular chimera *i*, respectively. *SortCount_wild type_* and *InputCount_wild type_* are the read counts for the different synonymous sequences encoding the human BTK SH2 domains included in the library, which are then averaged. A fitness value of 0 indicates that the chimera induces the same amount of CD69 expression as the wild-type BTK protein, negative values indicate reduced CD69 expression, and positive values indicate increased CD69 expression. Fitness scores from this assay agreed well among biological replicates (*SI Appendix*, Fig. S2 *A* and *B*, Pearson R ≥ 0.65 for n = 328 SH2 domain variants for four biological replicates in the Jurkat assay and Pearson R ≥ 0.62 for n = 328 SH2 domain variants for three biological replicates in the Ramos assay). Results from the Jurkat-cell and Ramos-cell experiments agreed, except where indicated below (*SI Appendix*, Fig. S2*C*, Pearson R = 0.60).

We also considered the possibility that differences in fitness between the BTK chimeras might be due to differences in protein abundance. To examine this, we tagged each chimeric BTK protein at its C-terminus with the mGreen-Lantern protein (a brighter GFP variant) (20). We then prepared lentiviruses from this library and transduced ITK-deficient Jurkat cells. We sorted the Jurkat cells by mGreen-Lantern fluorescence. The sorted and input populations were sequenced and the same fitness metric as used for CD69 upregulation was calculated, except that for this experiment we refer to this metric as “abundance” because it indicates the level of protein abundance from each SH2 chimera. Abundance measurements from this experiment showed good agreement among biological replicates (*SI Appendix*, Fig. S2*D*, Pearson R ≥ 0.62 for n = 328 SH2 domain variants for three biological replicates in the Jurkat assay). As expected, BTK proteins with SH2 domains containing stop codons exhibit strongly decreased abundance because the C-terminal mGreen-Lantern protein is not translated (*SI Appendix*, Fig. S2*E*).

### Phosphotyrosine Recognition by the SH2 Domain Is Required for Functional Chimeric BTK Proteins.

A phosphotyrosine-dependent interaction between the scaffold protein SLP65 (BLNK) and the BTK SH2 domain is important for triggering calcium signaling downstream of the B cell receptor in Ramos cells (16). The R307K mutation in the BTK SH2 domain disrupts this interaction because the arginine residue is critical for phosphotyrosine recognition (16–18, 21) (*SI Appendix*, Fig. S1*D*). We introduced arginine to lysine substitutions at positions corresponding to Arg 307 in BTK and 61 BTK chimeras that have at least 60% identity to the human BTK SH2 domain. Replacement of the arginine reduces the fitness of human BTK and most of the chimeras for which this mutation was tested (57 out of 61 in the Jurkat assay and 50 out of 61 in the Ramos assay, Fig. 1*D* and *SI Appendix*, Fig. S2*F*). For most of the chimeric BTK variants, substitution of the arginine for lysine at position 307 results in either no change in abundance or a modest decrease (Fig. 1*E*). The decrease in abundance is small compared to the decrease in fitness upon introducing the arginine to lysine substitution in SH2 domains even though stop codon substitutions result in similar decreases in fitness or abundance in both assays (Fig. 1*E*).

### Many SH2 Domains Increase BTK Fitness When Substituted for the BTK SH2 Domain.

A surprising result of measuring the fitness of BTK chimeras in Jurkat T cells is that many of the chimeric variants exhibit *increased* fitness compared to human BTK. For the four SH2 domains derived from the human Tec kinases (excluding BTK), SH2 domains from TXK and BMX increased fitness when swapped into human BTK (Fig. 1*F*). This trend holds when examining the 249 SH2 domains in the library (excluding controls), as about half (51%, or 128 sequences) had fitness scores greater than 0.

**Fig. 2.**
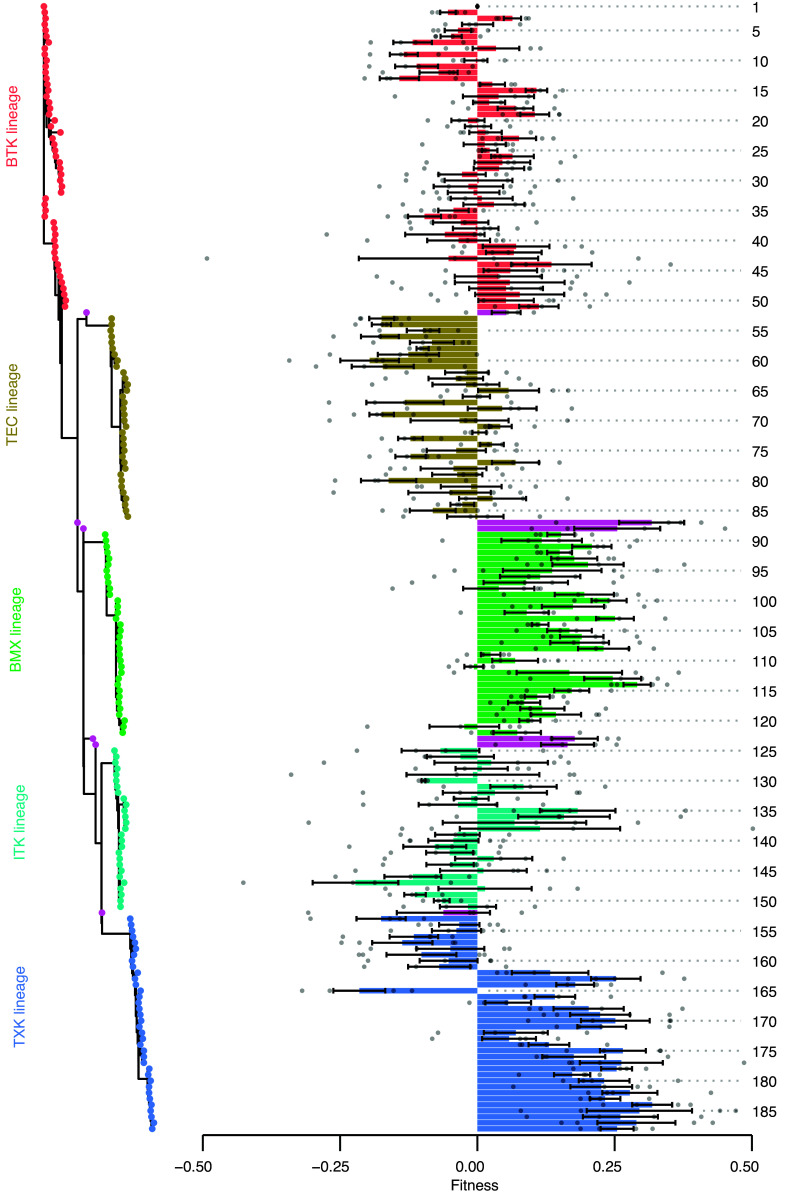
SH2-domain substitutions increase fitness in BTK. Fitness scores for the 188 SH2-domain chimeras corresponding to Tec kinases or ancestral SH2 domains are shown. SH2 domains are ordered by their sequence relationships. The fitness bars, measured using the Jurkat assay, are colored by the human Tec kinase to which they are most closely related, requiring at least 75% sequence identity to display that color (sequences without 75% sequence identity to a human Tec kinase are colored purple). Error bars represent SEM and points are the individual replicate values. Sequence numbering corresponds to *SI Appendix*, Table S1.

We constructed a phylogenetic gene tree for the Tec-derived SH2 domains and plotted the sequence relationships along with the fitness scores for each sequence (Fig. 2). The resulting data show that sequences cluster into groups that increase or decrease fitness similarly. Despite some measurement noise, it is clear that SH2 domains from two classes, corresponding to the BMX and TXK domains, increase fitness when substituted into human BTK. Similar experiments performed using the Ramos B cell assay also showed that the TXK and BMX classes of SH2 domains increased fitness when substituted into human BTK (*SI Appendix*, Fig. S3*A*). The BTK proteins with SH2 domains in the TXK or BMX classes do not have increased abundance overall, indicating that their increased fitness is not explained by increased protein stability or gene expression (*SI Appendix*, Fig. S4). Not all sequences show concordance between the T cell and the B cell assay results. In particular, 15 extant sequences in the TXK class exhibit fitness scores that strongly depend on the cell line (*SI Appendix*, Fig. S3*B*).

The c-Abl and c-Src SH2 domains, which are more distant in sequence from Tec-kinase SH2 domains, increase fitness when substituted into BTK (*SI Appendix*, Fig. S5*A*). However, substitution by even more distantly related SH2 domains tends to decrease fitness in general. Most of these distantly related SH2 domains (44 out of 54) decrease protein abundance when swapped into BTK (*SI Appendix*, Fig. S5).

### Using Ancestral Sequence Reconstruction to Focus on Fitness-Increasing Substitutions.

To determine why the BMX SH2 domains increase fitness of the chimeric BTK proteins, we used ancestral sequence reconstruction to focus attention on small sets of substitutions that modulate fitness. This approach has been used to examine mechanisms of drug selectivity in c-Src and c-Abl (22). The technique has also been used extensively in studies of evolutionary trajectories (23, 24). The human BMX and BTK SH2 domains differ at 41 positions that are distributed across the domain. We used ancestral-sequence reconstruction to generate SH2 sequences that fall in between those of BTK and BMX. BTK chimeras with these sequences exhibit intermediate fitness values (*SI Appendix*, Fig. S5 *B* and *C*).

**Fig. 3.**
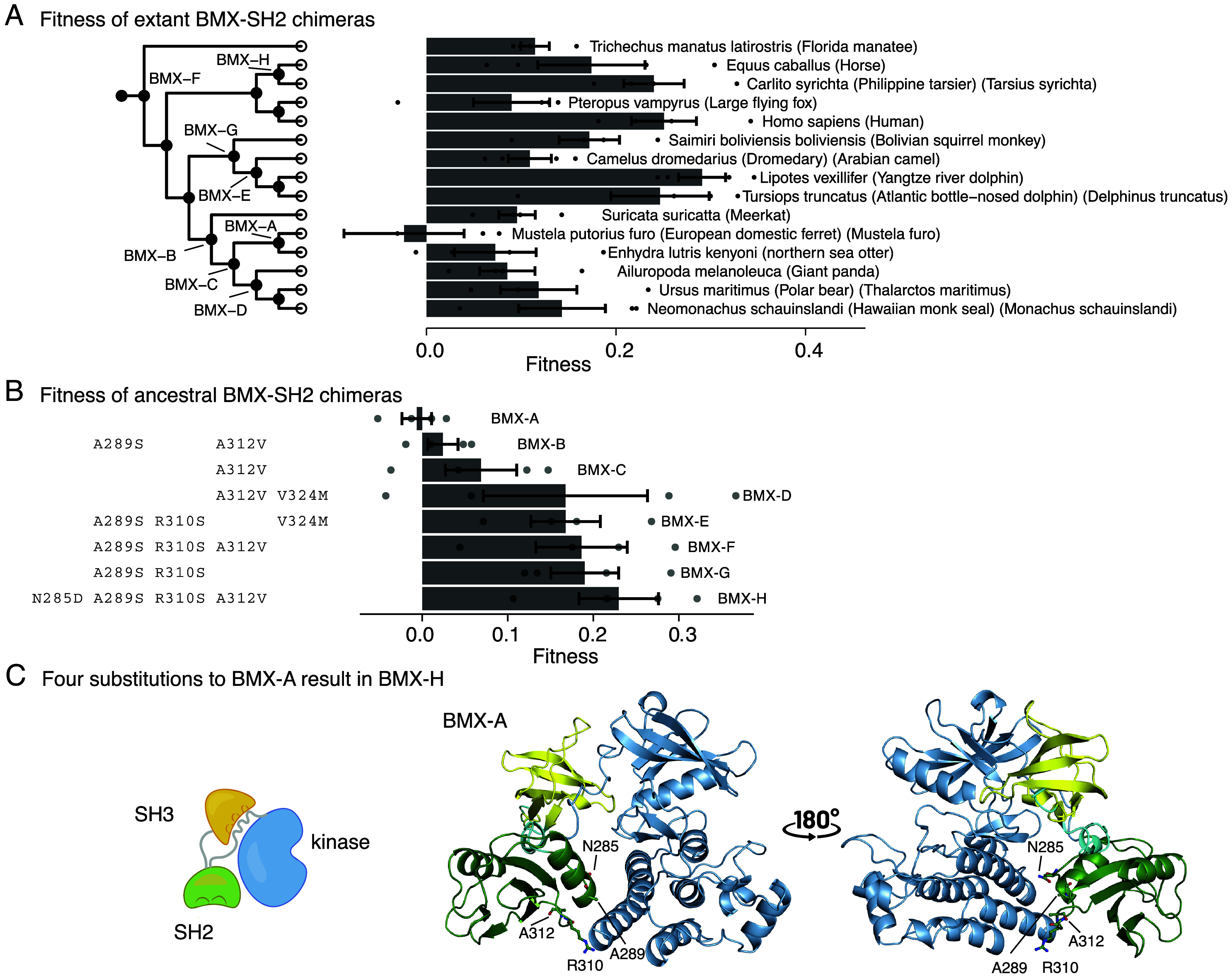
Ancestral-sequence reconstruction charts the fitness progression in the BMX Lineage. (*A*) Fitness scores from the BMX group of SH2 sequences. Each sequence is shown with its Latin name and common name. Positions correspond to sequence relationships based on a phylogenetic gene tree (*Left*), in which ancestors that were reconstructed are labeled. Error bars represent SEM. One point was removed from the *Mustela furo* bar at x = –0.2. (*B*) The eight ancestral-reconstructed sequences from (*A*), arranged by order of increasing fitness. The substitutions to the BMX-A sequence are shown at the *Left*. (*C*) An Alphafold (25) model of the Src module of the BMX-A sequence. The four residues (Asn 285, Ala 289, Ala 312, Arg 310) substituted in the BMX-H protein chimera are shown as a stick representation.

Eight reconstructed ancestral SH2 domains within the BMX group (denoted BMX-A through BMX-H) increase fitness progressively when substituted into human BTK (Fig. 3 *A* and *B*). The BTK chimera with the BMX-A SH2 domain (with 50% identity to the human BTK SH2 domain), behaves similarly to human BTK (fitness = 0.0 ± 0.017). In contrast, the BTK chimera with the BMX-H SH2 domain exhibits increased fitness (fitness = 0.23 ± 0.046). Between BMX-A and BMX-H, there are four substitutions in the SH2 domain: N285D, A289S, R310S, and A312V. The four residues that are substituted are not expected to interact with the phosphotyrosine residue in the target peptide (*SI Appendix*, Fig. S5*D*). These residues are located at the interface between the SH2 and kinase domains as seen in the mouse Btk crystal structure [PDB code: 4XI2 (10)], suggesting that disruption of stabilizing interactions between the SH2 and kinase domains may underlie the observed increases in fitness of the mutants (Fig. 3*C*).

### Kinase–Domain Mutations Decrease Fitness in a Manner That Depends on the SH2 Domain.

To explore further the role of the SH2-kinase domain interface in autoinhibition, we generated mutations in the kinase domain that alter this interface. We mutated helix αI^kinase^, the C-terminal helix in the kinase domain (*SI Appendix*, Fig. S6*A*), which interacts with the SH2 domain in the crystal structure of mouse Btk (10). We created a library of αI^kinase^ sequences from a multiple sequence alignment of 364 BTK sequences from the jawed vertebrates, curated by HomoloGene (26). 113 of these sequences were unique. We included the five human Tec kinase αI^kinase^ sequences for a total of 118 sequences, and swapped these sequences for αI^kinase^ in human BTK (*SI Appendix*, Fig. S6*B*).

We sought αI^kinase^ sequences that decrease the fitness of chimeric BTK by stabilizing the inactive conformation. The challenge in identifying such sequences is that sequences that destabilize the protein are also expected to decrease fitness. To filter out destabilizing sequences, we measured the fitness of αI^kinase^ variants in two genetic backgrounds: wild-type BTK and the gain-of-function BMX-H SH2 chimera. An αI^kinase^ variant that destabilizes the kinase domain should reduce fitness in both BTK and the BMX-H SH2 chimera (*SI Appendix*, Fig. S6*C*). In contrast, an αI^kinase^ variant that reduces fitness by making interactions that stabilize the BTK SH2-kinase interface should reduce fitness in BTK, but have little or no effect in the BMX-H SH2 chimera, which has a different SH2 domain (*SI Appendix*, Fig. S6*C*).

We developed a barcoding approach to determine the identity of the SH2 domain and the αI^kinase^ variant in high-throughput assays simultaneously (*SI Appendix*, Fig. S7*A*). The αI^kinase^ variants were cloned into human BTK and into BTK variants with the BMX-H SH2 domain. The *BTK* genes also contained a three-nucleotide barcode after the stop codon that was associated with the SH2 domain. One sequencing read was used to determine both the identity of αI^kinase^ and the three-nucleotide barcode, which identified the SH2 domain. The resulting fitness scores from these libraries were reproducible among biological replicates (R ≥ 0.87 for n = 236 proteins and n = 4 biological replicates, *SI Appendix*, Fig. S7*B*). The fitness scores showed good agreement between the Jurkat T cell assay and the Ramos B cell assay (*SI Appendix*, Fig. S7*C*, Pearson R = 0.68). In keeping with a previous study (13), substitution of Asp 656 in αI^kinase^ increased fitness (*SI Appendix*, Fig. S7*D*).

**Fig. 4.**
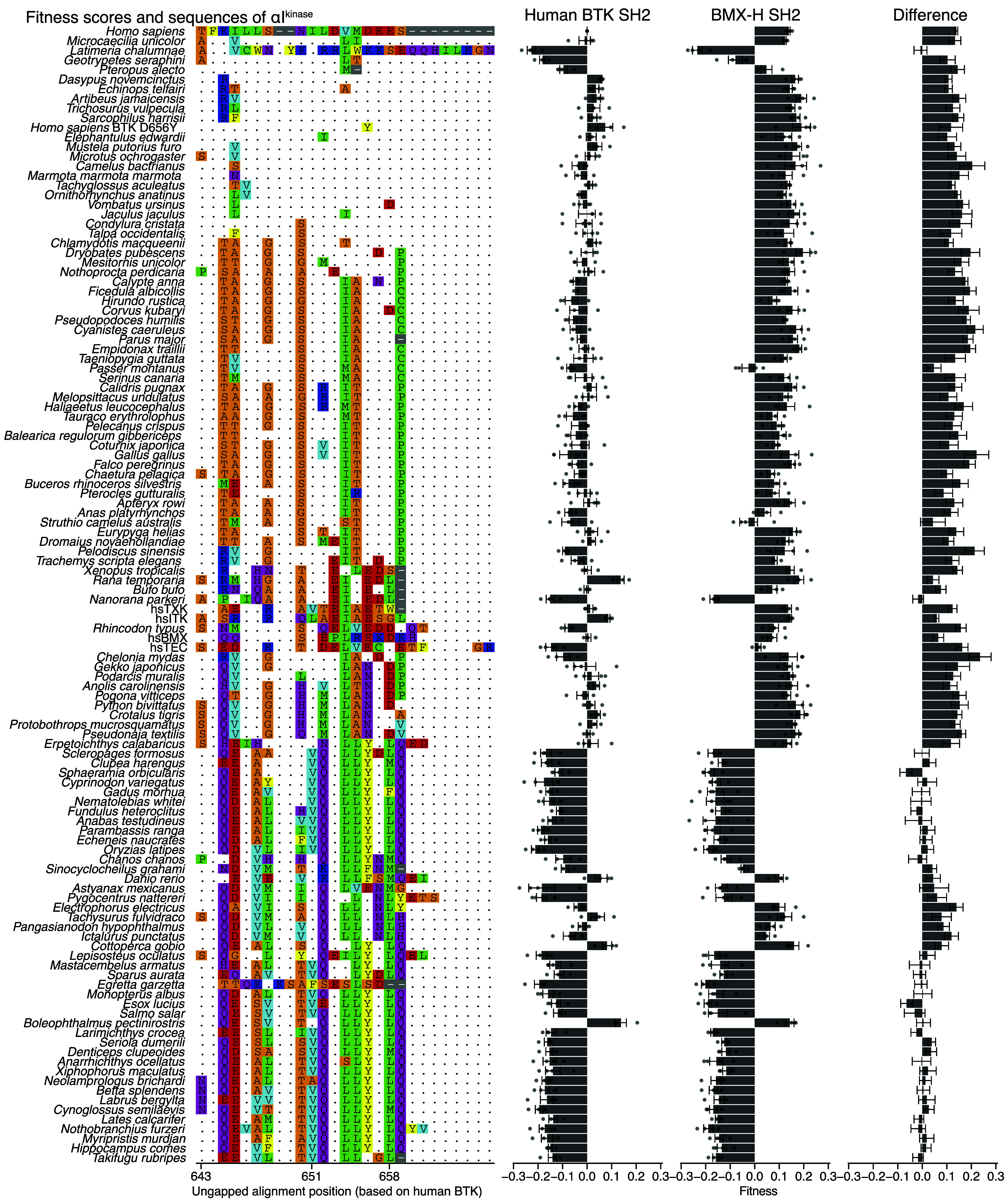
Screening αI^kinase^ substitutions. The 118 αI^kinase^ sequences are shown along with a multiple sequence alignment and fitness scores. The fitness scores are shown for the human BTK SH2 genetic background, the BMX-H SH2 genetic background, and the difference between these two backgrounds (BTK values subtracted from the BMX-H values). Error bars represent SEM and points represent the individual replicate values. For the difference values, error bars are determined using the SE propagation formula.

**Fig. 5.**
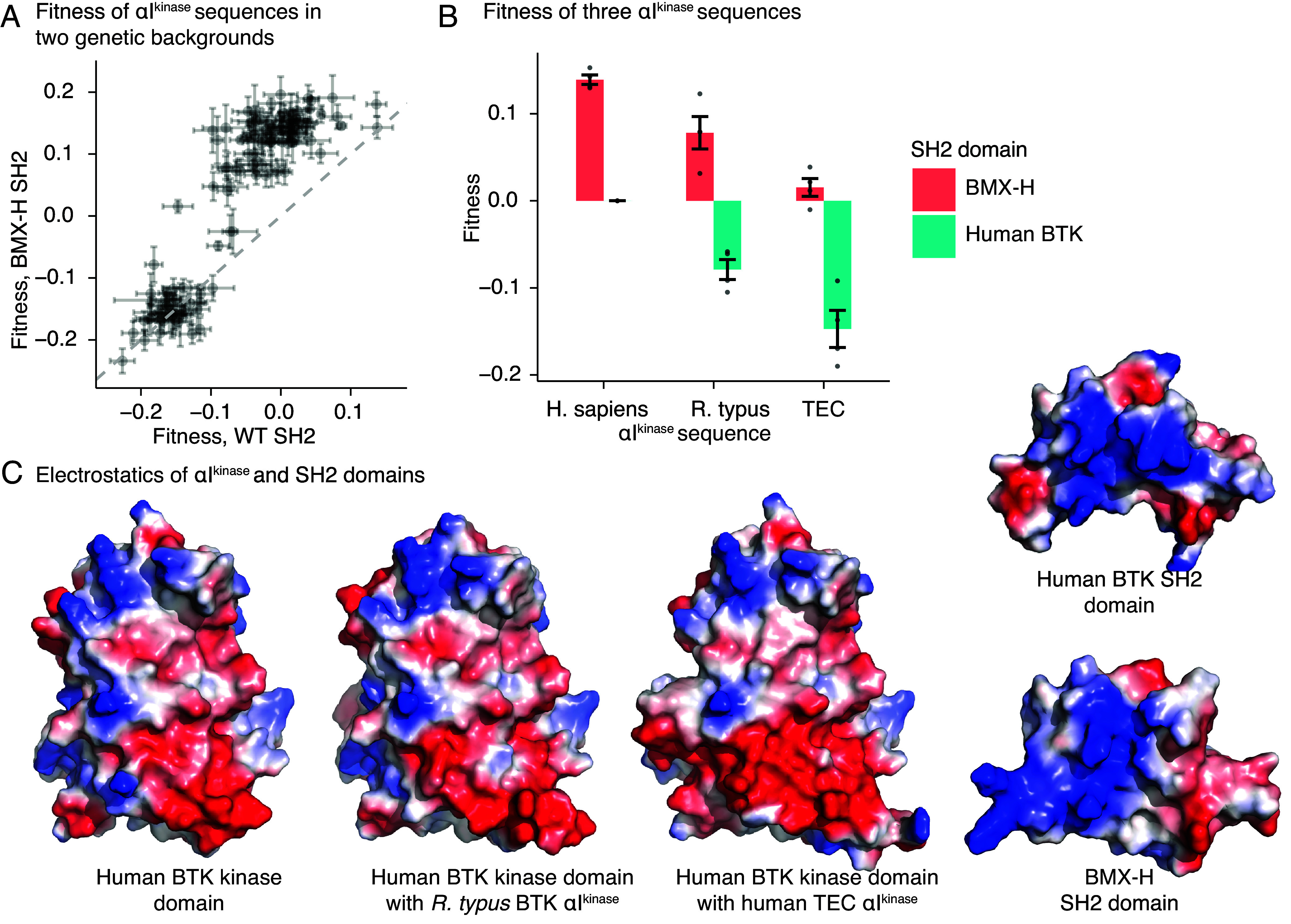
Two αI^kinase^ substitutions decrease fitness consistent with enhanced autoinhibition. (*A*) Fitness scores from the 118 αI^kinase^ sequences shown in two different genetic backgrounds. Error bars represent SEM. (*B*) Fitness scores from three αI^kinase^ sequences (*x* axis) shown in two genetic backgrounds (key). (*C*) Alphafold (25) model of the human BTK protein, the αI^kinase^ sequence of *Rhincodon typus* in the human BTK kinase domain, and the αI^kinase^ sequence of TEC in the human BTK kinase domain. The kinase–domain interacting interfaces of the human BTK and BMX-H SH2 domains are shown at the *Right*. Coloration is based on charge, with positively charged regions in blue and negatively charged regions in red.

As expected, many αI^kinase^ sequences exhibit negative fitness scores in both the human BTK and the BMX-H backgrounds, consistent with destabilization of the kinase domain (Figs. 4 and 5*A*). For example, Ile 651 in αI^kinase^ forms a hydrophobic packing interaction with the adjacent helix, αG^kinase^, in the C-lobe of the kinase domain (*SI Appendix*, Fig. S7*E*). Substitution of Ile 651 by the smaller valine residue (for example, see *Salmo salar*, arctic salmon, and *Boleophthalmus pectinirostris*, great blue spotted mudskipper, Fig. 4) results in loss of fitness regardless of the identity of the SH2 domain.

We identified two αI^kinase^ sequences, derived from human TEC and from *Rhincodon typus* (whale shark) BTK, that decreased fitness in human BTK but not in the BMX-H SH2 chimera (Fig. 5*B*). Structural models of the human BTK kinase domain with *R. typus* or TEC αI^kinase^, generated by Alphafold (25), show an increase in net negative charge on αI^kinase^ that parallels the fitness values of the corresponding chimeras (Fig. 5*C* and *SI Appendix*, Fig. S7*F*). The kinase-binding region of the BTK SH2 domain has a more positive electrostatic potential than the corresponding region of the BMX-H SH2 domain (Fig. 5*C*). Thus, the two αI^kinase^ variants, with one and two additional negative charges each, are consistent with a mechanism in which enhanced electrostatic interaction between the SH2 and kinase–domain interfaces decreases fitness, presumably through stabilization of the autoinhibited conformation.

## Discussion

In this study, we examined the effect of replacing the SH2 domain of BTK with hundreds of SH2 domains from other proteins. Most of the chimeric BTK proteins that we tested are functional substitutes for human BTK. Surprisingly, we found that many of the chimeras exhibited *increased* fitness with respect to human BTK. For one set of chimeric proteins, we traced these fitness increases to disruption of the SH2-kinase domain interface seen in the structure of inactive Btk (10), confirming that the SH2 domain is autoinhibitory even though BTK lacks an obvious latching mechanism. The observation that many of these results are reproduced in Ramos cells, which do not depend on BTK kinase activity (14, 27), suggests that disruption of the autoinhibited conformation enhances the scaffolding functions of BTK. This might stem from an increased ability of the SH2, SH3, and kinase domains to interact with other molecules when not stabilized in the autoinhibited conformation.

The nature of the autoinhibitory interface between the SH2 domain and the kinase domain is predominately electrostatic, with negatively charged residues in the C-terminal helix of the kinase domain (αI^kinase^) located near positively charged residues in the phosphotyrosine-binding site of the SH2 domain. Indeed, phosphopeptide binding and SH2-kinase docking appear mutually exclusive (13). The autoinhibitory interactions are not supported by many closely related SH2 domains, and the substitution of these SH2 domains into BTK increases signaling. Thus, the targeting function of the SH2 domain is readily transferable, while the inhibitory function is not.

Our results suggest that in a prototypical Src module without a latching mechanism, distributed interactions between the SH2 domain and the kinase domain may have sufficed to suppress kinase activity and impede the targeting function of the SH2 domain until released by activation of the kinase domain. Studies on the Src-family kinase HCK show that the majority of HCK molecules adopt the compact assembled conformation even without C-terminal tail phosphorylation, suggesting that Src-family kinases, like BTK, have evolved a complementary interface between the SH2 and kinase domains that favors autoinhibition (28). Alteration of this interface in the viral oncoprotein v-Src may also contribute to its constitutive activity. v-Src isolated from *Gallus gallus* (chicken) contains a leucine residue in αI^kinase^ that replaces a glutamate residue (Glu 517) in chicken c-Src. In c-Src, the glutamate makes an ion pair with an arginine in the SH2 domain, but v-Src lacks this interaction, which may destabilize the SH2-kinase interface and shift v-Src toward the active state.

Our results highlight the importance of the SH2-kinase interface in an ancient switching mechanism built into the Src module. Kinases with this simple switching mechanism are seen in the choanoflagellates, and therefore arose before the establishment of the metazoan lineage (29). These kinases have survived to generate some of the most important signaling proteins in humans.

## Methods

### Ancestral Sequence Reconstruction.

All 59,304 SH2 domain sequences in the PFAM database were downloaded as a multiple sequence alignment. These domains align to the human BTK SH2 domain, corresponding to residues 281 to 362. After deduplication, this set contained 23,121 SH2 domains, of which 156 were derived from human proteins. This set of human SH2 domains was filtered to include only the 66 SH2 domains that had at least 25% pairwise identity to the human BTK SH2 domain. This set was then divided into Tec-kinase SH2 domains (those with ≥40% identity to the human BTK SH2 domain) and more distantly related SH2 domains (those with identity to the human BTK SH2 domain between 25% and 40%). The six human Tec SH2 domains consisted of TEC, ITK, TXK, BMX, Q5JY90 (an isoform of BTK), and BTK. Note that although Q5JY90 was included in the library preparation, it was not analyzed for this study and therefore is not included in the discussions of the library.

The six human Tec SH2 domains were then used to mine the original set of unique PFAM domains for an additional set of 84 nonhuman Tec-kinase domains. These domains were selected from other organisms by progressively choosing 14 domains for each Tec-kinase sequence with increasing pairwise identity from the human Tec-kinase domain, excluding any domains with a single mismatch to the human domain. The resulting set of 90 domains included 15 domains for each of the six Tec-kinase SH2 domains.

These Tec-kinase domains from humans and other organisms were used for ancestral sequence reconstruction. (One domain, A0A452S617, the Q5JY90 BTK isoform from the American black bear, was removed from the ancestral sequence reconstruction because of low quality). First, a maximum-likelihood tree was generated using Practical Alignment using Saté and TrAnsitivity algorithm (30). Then the PAML software suite [version 4.8a, August 2014 (15)] was used to generate ancestral sequences. To perform this reconstruction, we implemented both marginal (31) and joint (32) reconstruction of the ancestral sequence. Marginal reconstruction is optimizing the state at the current node that maximizes the probability over the entire tree. Joint reconstruction is optimizing all states simultaneously to maximize the probability over all nodes. The model used the SWISSPROT probabilities of exchanging amino acids (33).

PAML reconstruction uses all positions in all sequences in the alignment and cannot determine ancestors from sequence alignments with gaps. We reasoned that regions with gaps would generally be variable regions that could be important for targeting specificity or kinase–domain interaction and chose two methods to reconstruct these gap sequences. In the first method, any position that contained at least one gap in the alignment was removed from the alignment entirely, resulting in all sequences aligning to the shortest sequence. In the second method, residues were included at all gaps, interpolating from all other sequences in the alignment. This method aligns all domains to the longest sequence. We implemented both methods to reconstruct ancestral nodes. This analysis resulted in 114 ancestral nodes. In Fig. 2 and *SI Appendix*, Table S1, a prime (′) denotes sequences that were constructed from the longest domain and those without a prime were constructed from the shortest domain. The resulting nodes were combined with the extant Tec (90 domains) and more distant domains (60 sequences) for a total of 264 domains. This set of domains was further filtered to remove ancestral nodes from the Q5JY90 lineage (nine nodes) and domains longer than 88 amino acids (six sequences) for a final set of 249 domains. Extant domains corresponding to Q5JY90 were excluded because they have a large truncation in the SH2 domain and decrease fitness.

Control sequences were added to this set to determine the effect of disrupting the phosphotyrosine binding residue (R307K substitutions) or of completely removing BTK by adding a stop codon (R307X). We reasoned that these controls would only be informative for sequences like the wild-type human BTK sequence, as very distant domains, even if they increased fitness, would be more difficult to interpret the effects of phosphotyrosine targeting. Any domain of the set of 249 (including ancestral domains) with a pairwise identity greater than 60% to the human BTK SH2 domain (62 domains, including BTK) was included with the R307K mutation. A subset of these 62 domains with pairwise identity to the human BTK SH2 domain of greater than 95% (17 domains) were also mutated to contain a stop codon at position 307 to determine the baseline fitness in the assay. All 79 control sequences were included with the original 249 sequences for a total of 328 SH2 domain sequences. Fig. 2 and *SI Appendix*, Figs. S3*B* and S5*A*, which paired the fitness data with a phylogenetic tree, were constructed using the ggtree plotting software (34).

### Reverse Translation of Sequences.

Nucleotide sequences were constructed from each of the 328 protein sequences by generating four synonymous nucleotide sequences for each protein sequence. If an amino acid in a protein sequence was identical to that of BTK, the human BTK codon was used at that position. Otherwise, a codon was chosen at random, excluding sequences that contained the BsaI recognition sequence. 31 additional codon variants of the wild-type human BTK SH2 sequence were included by choosing five codon positions at random and substituting a synonymous codon. In the αI^kinase^ library, 44 synonymous wild-type sequences were included. Handles allowing for BsaI digestion were then included at each end (left handle: 5′ CAGGCATGGTCTCATGAG 3′; right handle 5′ CAGCAGAGACCCGATTGG 3′). Because the resulting nucleotide sequences were different lengths depending on the length of the SH2 domain, sequences were padded with additional sequence from the PhiX genome for a final length of 300 nt. Sequences were ordered from Twist Biosciences as oligo pools.

### High-Throughput Cloning.

The pooled oligos of SH2 domain sequences were amplified by 15 cycles of PCR using primers that annealed to the left and right constant sequences (oligos #272 and #273, *SI Appendix*, Table S2). BTK constant sequences corresponding to gene segments 5′ of the SH2 domain and 3′ of the SH2 domain were also amplified in separate reactions using a BTK template in which endogenous BsaI restriction sites were removed by replacing them with synonymous codons. These constant segments were amplified with oligos #274 and #275 for the 5′ segment, and oligos #276U and #277 for the 3′ segment (*SI Appendix*, Table S2). The left oligo of the 5′ segment encodes a XmaI restriction site while the right oligo of the 3′ segment encodes a BamHI restriction site.

The three pieces of BTK (corresponding to the amplified pool of SH2 domains, the 5′ constant BTK segment, and the 3′ constant BTK segment) were agarose-gel purified and combined in a 50 µL reaction containing 2 µL of BsaI HF (New England Biolabs), 2 µL of T4 DNA ligase (New England Biolabs), 5 µL of 10× T4 DNA ligase buffer (New England Biolabs) and 0.5 µL of DpnI (New England Biolabs). The DpnI was intended to remove any contaminating uncut vector, which was the template in the PCR of the constant sequences. This ligase mixture was incubated for 2 h at 37 °C and then overnight cycling between 12 °C for 10 s and 37 °C for 10 min. The reaction was run on a 1% agarose gel and the fully ligated product (~2 kb) was excised and purified.

The assembled BTK sequence was digested with BamHI HF (New England Biolabs) and XmaI (New England Biolabs) in a 30 µL reaction containing 3 µL of CutSmart buffer (New England Biolabs). The digested sequence was ligated into a vector backbone (Addgene #21373) containing an internal ribosome entry site (IRES) followed by enhanced green fluorescent protein (EGFP) that had been digested with BamHI, XmaI, and EcoRI (EcoRI digests the BTK gene, and is included when preparing the vector backbone to decrease contamination from uncut human BTK). The ligation reaction used a 5 to 1 insert to vector ratio and 100 ng of vector. After 1 h at room temperature, the ligation reaction was purified using the Zymo Clean and Concentrator kit (Zymo Research) according to the manufacturer’s instructions and DNA was eluted in 6 µL. 3 µL of the elution was electroporated into Endura electrocompetent cells (Lucigen) according to the manufacturer’s instructions. Cells were recovered in 1 mL of Recovery Media (Lucigen) for 1 h at 30 °C and diluted to 50 mL in Luria Broth. 50 µL of this dilution were plated as a diagnostic to gauge transformation efficiency, along with a no-insert control. The remaining volume was grown overnight at 30 °C. In the morning, plasmid DNA was extracted from the entire sample volume using a midiprep kit (Qiagen) according to the manufacturer’s instructions.

The αI^kinase^ library was prepared in two vector backbones. First, the BMX-H SH2 domain was used to replace the human SH2 domain in the BTK sequence. The BMX-H SH2 sequence was purchased from Twist Bioscience as a gene fragment, which was then amplified using oligos #468 and #469 (*SI Appendix*, Table S2). The amplicon was ligated to the 5′ and 3′ arms of BTK using the ligation scheme above, and then the BamHI/XmaI fragment was ligated into the original digested pHIV vector. The αI^kinase^ library was purchased as an oligo pool from Twist Bioscience and amplified in four separate PCRs using the universal forward primer (oligo #432) and one reverse oligo (#433, #434, #435, or #436, *SI Appendix*, Table S2). These reverse oligos included a three-nucleotide barcode after the stop codon and the BamHI restriction site. Two 5′ constant sequences were then prepared by amplifying either the human BTK sequence or the BMX-H SH2 chimera, both with oligos #437 and #275, but using their respective templates. These constant sequences were then ligated to the barcoded, amplified αI^kinase^ library by matching oligos #433 (barcode TTC) and #434 (barcode CCT) to the human SH2 domain and oligos #435 (barcode GGA) and #436 (barcode AAG) to the BMX-H SH2 domain. Ligation reactions were cloned into the pHIV backbone as above, the sample was purified using a Midiprep kit (Qiagen), and then the resulting plasmid libraries were pooled prior to lentivirus preparation.

The library of SH2 chimeras used for abundance was cloned using the same method as the fitness library but cloned into a plasmid that contained mGreen-Lantern, rather than IRES-EGFP. The insert was prepared exactly as described for the fitness library, digested with BamHI/XmaI, and ligated to a backbone that contained an in-frame mGreen-Lantern protein tag at the 3′ end of the coding sequence. Between BTK and mGreen-Lantern, a linker with sequence ATLYNKVGGGGS was included. The pHIV plasmid backbone was also used for these libraries.

### Lentivirus Preparation and Titering.

Lentiviruses were prepared as previously described (14). Briefly, HEK293FT cells were seeded at a density of 250,000 cells/mL in 5 mL in 6-cm dishes in culture media (day 1). The next day (day 2), cells were transfected with a mixture of transfer plasmid containing the SH2-BTK chimeras or the αI^kinase^ chimeras (modified from pHIV-EGFP, Addgene #21373, 5 µg) and two packaging plasmids (pMDG.2, Addgene #12259, 1.25 µg and psPAX2, Addgene #12260, 3.75 µg) in 500 µL of opti-mem (Thermo Fisher) using Lipofectamine LTX (Thermo Fisher) according to the manufacturer’s instructions. The following morning (day 3) cells were refed. The following day (day 4) 5 mL of viral supernatant was harvested and stored at 4 °C, and the cells were refed. The following day (day 5) an additional 5 mL of viral supernatant was harvested and combined with the previous day’s aliquot for a total of 10 mL of viral supernatant. This was then concentrated using Lenti-X concentrator (Takara Bio) according to the manufacturer’s instructions, and the pellet from this concentration was resuspended in 1 mL of Roswell Park Memorial Institute (RPMI) medium + 5% fetal bovine serum, aliquoted, flash frozen in liquid nitrogen, and stored at –80 °C.

Lentiviruses were titered by thawing an aliquot of virus and adding 0, 5, 10, 20, 40, or 80 µL of virus to 500 µL ITK-deficient Jurkat T cells or BTK-deficient Ramos B cells at 500,000 cells/mL in a 24-well plate, along with 4 µg/mL polybrene. The following day, cells were centrifuged (300 g for 5 min) and resuspended in 1 mL of fresh media, then plated onto a 96-well plate. Cells were grown for two additional days and then the plate was analyzed for GFP fluorescence using an Attune cell analyzer (Thermo Fisher) equipped with a 96-well plate autosampler. The fraction of GFP-positive cells as a function of concentration was fit to the standard binding isotherm to obtain titer values.

### Cell Culture.

All cells used in this study were cultured in a humidified incubator at 37 °C with 5% CO_2_. BTK-deficient Ramos B cells were cultured in RPMI (Thermo Fisher) containing 10% fetal bovine serum (Thermo Fisher) and 1× Glutamax (Thermo Fisher). ITK-deficient Jurkat cells (35) were cultured in RPMI containing 5% fetal bovine serum and 1× Glutamax. HEK293FT packaging cells, used for lentivirus preparation, were cultured in Dulbecco’s Modified Eagle Medium (Thermo Fisher) containing 10% fetal bovine serum and 1× Glutamax. All cell lines tested negative for *Mycoplasma* contamination.

### High-Throughput Assays.

High-throughput experiments were performed as described (14). Briefly, ITK-deficient Jurkat T cells or BTK-deficient Ramos B cells were plated in 10 mL at a density of 500,000 cells/mL in 10 cm dishes in triplicates or quadruplicates. The reason that both versions of the assay are used is that BTK kinase activity is required for CD69 expression in the Jurkat assay but kinase activity is dispensable in the Ramos assay. The Ramos assay thus provides clues as to the structural features important for the kinase-independent functions of BTK. The cells were then transduced on day 1 with titered lentiviral libraries, adding a volume of virus such that at most 25% of the cells were GFP positive (corresponding to <3% of cells having a multiplicity of infection > 1) and 10 µg/mL polybrene.

The following morning (day 2) cells were refed by centrifugation at 300 g for 5 min followed by resuspension in fresh media, with a 2× dilution, and replated. Cells were allowed to recover for one additional day (day 3). On day 4, Ramos cells were stimulated in the evening with 4 µg/mL antibody targeting IgM (I2386, Sigma-Aldrich).

On day 5, Ramos or Jurkat cells were stained with antibody targeting CD69 as follows. Cells were concentrated by centrifugation at 300 g for 5 min, then resuspended in 200 µL of cell-staining buffer (phosphate-buffered saline containing 10% FBS and 0.05% w/v sodium azide) containing a 1:20 dilution of PerCP-Cy5.5 conjugated antibody targeting CD69 (Cell Signaling Technology #28633). Cells were then incubated on ice for 30 min in the dark. Following this incubation, cells were centrifuged again and washed with 500 µL cell-staining buffer, then centrifuged and resuspended in 1 mL of cell-staining buffer for sorting. After this staining protocol, 100 µL (10%) of sample was removed as input and kept on ice during sorting. Cells were sorted for a GFP-positive and CD69-positive population on a SH800 Cell Sorter (Sony) or FACSAria Fusion (BD Biosciences).

Gating was performed as follows. All cells were first gated based on forward and side scatter for a healthy population, generally 80 to 90% of the total cell number. To set the gates for the transduced (GFP-positive) and CD69 (PerCP-Cy5.5) positive populations, negative controls that were untransduced (GFP-negative) or unstained (PerCP-Cy5.5-negative) were used. Gates were set such that fewer than 0.1% negative-control cells fell within the positive gates. The double-positive population was then sorted for each library. In the case of Jurkat and Ramos cells, this double-positive population was approximately 5% of the total number of cells. Ramos cells required stimulatory antibody targeting IgM to express enough CD69 to sort 5% double-positive cells, while Jurkat cells did not require any stimulation. Each experiment sorted at least 200× the number of cells as variants in the library.

### Library Preparation and Sequencing.

Following sorting, input and sorted samples were mixed with 1 mL of TRI reagent (Sigma-Aldrich), concentrating the sorted sample by centrifugation (300 g, 5 min) prior to lysis if the volume exceeded 200 µL. RNA was extracted according to the manufacturer’s protocol, except that after the aqueous phase was transferred to a new tube, it was mixed 1:1 with chloroform and centrifuged at 21,000 g for 10 min at 4 °C as an additional wash step. 4 µL of linear acrylamide (Thermo Fisher) was added as carrier.

RNA-seq libraries were prepared from TRI reagent-extracted samples as follows, beginning with the reverse-transcription. Following precipitation, RNA was resuspended in water containing 5 µM RT primer (oligo #296, *SI Appendix*, Table S2). The mixture was heated to 65 °C for 5 min, then snap cooled on ice. The RNA–oligo mixture was then mixed with 1× First-Strand Buffer (Thermo Fisher), 0.5 mM deoxynucleotide triphosphates, 10 mM DTT, 1 µL SuperaseIn (Thermo Fisher), and 0.5 µL Superscript III (Thermo Fisher). The final reaction mixture was incubated at 50 °C for 1 h. Following this incubation, RNA was hydrolyzed by addition of 5 µL of 1 M NaOH and incubation at 90 °C for 10 min. The mixture was then neutralized with 25 µL of 1 M HEPES, pH 7.4, and desalted using a Micro Bio-Spin P-30 tris column (BioRad), eluting the cDNA in 60 µL.

The cDNA was PCR amplified in two rounds. The first-round PCR added the priming regions for Illumina sequencing but did not add the barcodes (using oligos #278 and #279 for the SH2 library and oligos #438 and #439 for the αI^kinase^ library, *SI Appendix*, Table S2). The second-round PCR used primers that annealed to the Illumina priming regions, adding the flow-cell hybridizing sequences and barcodes (using primers BC_p5_1, BC_p5_2, BC_p5_3, BC_p5_4, BC_p5_5, BC_p5_6, BC_p5_7, BC_p5_8, BC_p7_1, BC_p7_2, BC_p7_3, BC_p7_4, BC_p7_5, BC_p7_6, BC_p7_7, BC_p7_8, BC_p7_9, BC_p7_10, BC_p7_11, and BC_p7_12, *SI Appendix*, Table S2). Amplified libraries were gel-purified on a 2% agarose gel, extracted with a gel-extraction kit (Qiagen), pooled at equal molar based on the band intensities, and then concentrated using a Clean and Concentrator kit (Zymo). Libraries were sequenced using a MiSeq sequencer and V2 chemistry with paired-end 150 by 150 bp reads.

### Data Analysis.

Quantification of fitness from sequencing data was performed as previously described (14). Briefly, Fastq files from MiSeq runs were aligned to the Fasta files containing the full sequences of each variant using Kallisto (36) to generate read counts for each variant. A read cutoff of 50 reads was applied to the input libraries such that any variant not passing this threshold was discarded. Next, the unnormalized scores were calculated by dividing the number of reads in the sorted dataset by the number of reads in the input dataset and taking the base-10 logarithm. These unnormalized scores were normalized by subtracting the mean of the human BTK SH2 fitness scores, according to Eq. **1**:

[1] $$\begin{array}{c} {\mathrm{Fitness}}_{i}=\log_{10}\left(\frac{{\mathrm{SortCount}}_{i}}{{\mathrm{InputCount}}_{i}}\right)-\langle \log_{10}\left(\frac{{\mathrm{SortCount}}_{wild\;type}}{{\mathrm{InputCount}}_{wild\;type}}\right)\rangle . \end{array}$$

In Eq. **1**, *Fitness_i_* denotes the fitness score for a particular variant *i*. Because each sequencing library contained synonymous codon sequences of the human BTK protein sequence (rather than one human BTK sequence), fitness scores were calculated by subtracting the mean of these synonymous sequences.

Code to generate SH2-domain and αI^kinase^ libraries, perform ancestral-sequence reconstruction, and analyze RNA-seq libraries was written using R (37) and Python and is available on Github (https://github.com/timeisen/MutagenesisPlotCode and https://github.com/timeisen/SH2s).

## Supplementary Material

Appendix 01 (PDF)

Dataset S01 (TXT)

Dataset S02 (XLSX)

## Data Availability

Code for generating saturation-mutagenesis libraries and analyzing them is available on GitHub [https://github.com/timeisen/MutagenesisPlotCode (38) and https://github.com/timeisen/SH2s (39)]. Data have been deposited in DRYAD (https://datadryad.org/dataset/doi:10.5061/dryad.rxwdbrvjx) (40).
