# Supplementary material for: BTK autoinhibition analyzed by high-throughput swaps of SH2 domains
Source: Proc Natl Acad Sci U S A. 2025 Oct 10;122(41):e2502688122. doi: 10.1073/pnas.2502688122 (PMC12541323; doi:10.1073/pnas.2502688122)
Supplement: Supplementary file 1 — Appendix 01 (PDF) [file pnas.2502688122.sapp.pdf]

Supplementary Figures

Fig. S1. Design of the SH2-substitution experiment.
Fig. S2. Reproducibility of fitness and abundance scores.
Fig. S3. Comparison between fitness scores in Jurkat and Ramos cells.
Fig. S4. Comparison between the fitness and abundance scores for SH2-domain chimeras.
Fig. S5. Substitution of SH2 domains from distantly related proteins.
Fig. S6. Substitution of αI^{kinase}.
Fig. S7. Library construction of the αI^{kinase} sequences in two genetic backgrounds.

Figure S1

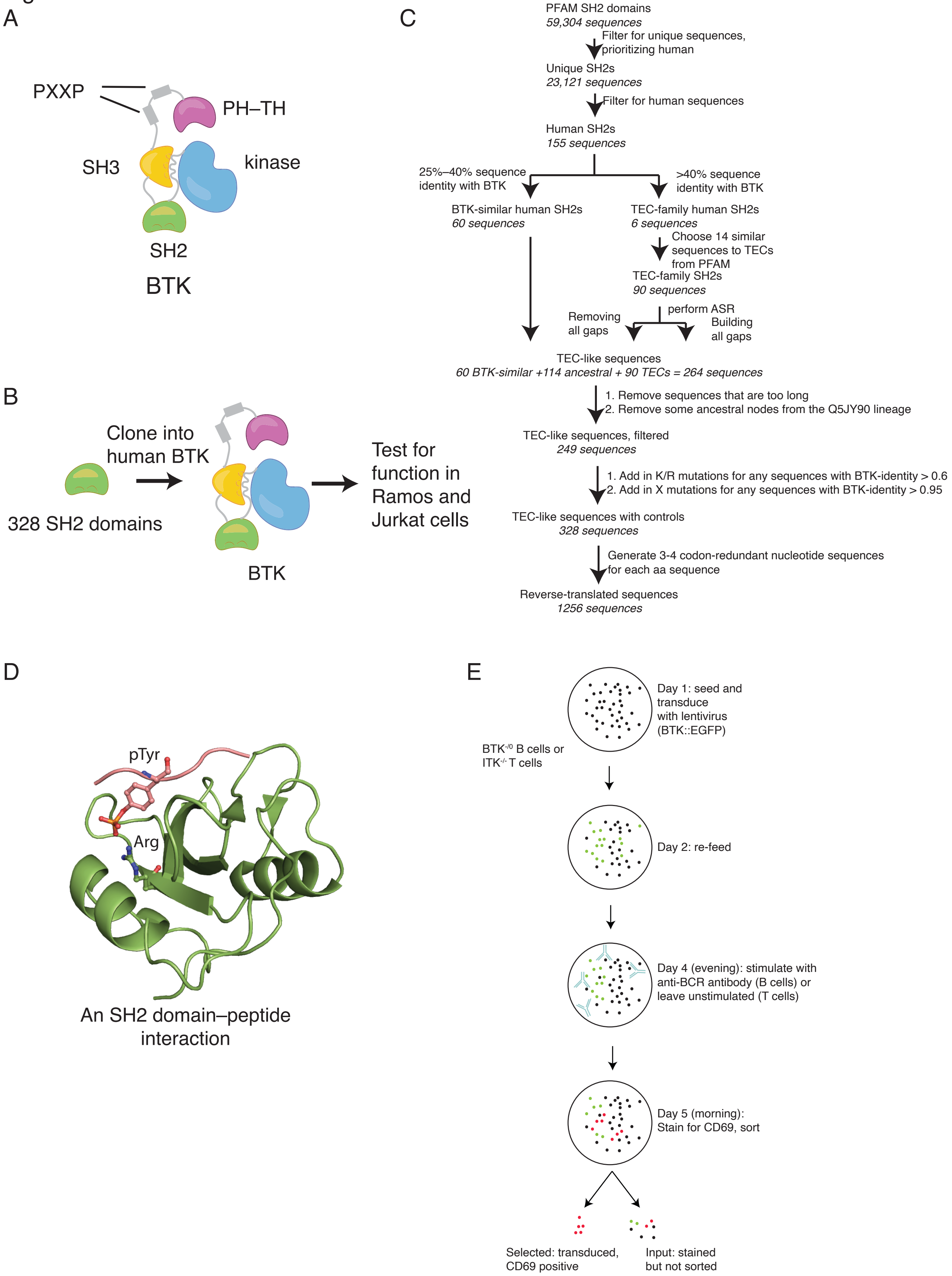
A
PXXP
PH–TH
SH3
kinase
SH2
BTK
B
Clone into human BTK
328 SH2 domains
BTK
Test for function in Ramos and Jurkat cells
C
PFAM SH2 domains
59,304 sequences
Filter for unique sequences, prioritizing human
Unique SH2s
23,121 sequences
Filter for human sequences
Human SH2s
155 sequences
25%–40% sequence identity with BTK
>40% sequence identity with BTK
BTK-similar human SH2s
60 sequences
TEC-family human SH2s
6 sequences
Choose 14 similar sequences to TECs from PFAM
TEC-family SH2s
90 sequences
perform ASR
Removing all gaps
Building all gaps
TEC-like sequences
60 BTK-similar +114 ancestral + 90 TECs = 264 sequences
1. Remove sequences that are too long
2. Remove some ancestral nodes from the Q5JY90 lineage
TEC-like sequences, filtered
249 sequences
1. Add in K/R mutations for any sequences with BTK-identity > 0.6
2. Add in X mutations for any sequences with BTK-identity > 0.95
TEC-like sequences with controls
328 sequences
Generate 3-4 codon-redundant nucleotide sequences for each aa sequence
Reverse-translated sequences
1256 sequences
D
pTyr
Arg
An SH2 domain–peptide interaction
E
Day 1: seed and transduce with lentivirus (BTK::EGFP)
BTK$^{-/0}$ B cells or ITK$^{-/-}$ T cells
Day 2: re-feed
Day 4 (evening): stimulate with anti-BCR antibody (B cells) or leave unstimulated (T cells)
Day 5 (morning): Stain for CD69, sort
Selected: transduced, CD69 positive
Input: stained but not sorted

Fig. S1. Design of the SH2-substitution experiment. (A) BTK, with all domains labeled. (B) Schematic of the experiment. (C) Flow chart describing the computational steps to choose SH2 domains to substitute in human BTK. (D) The SH2 domain, shown in cartoon representation in green, interacting with a phosphopeptide, in red. Arg 307 in human BTK, the conserved residue that interacts with the phosphotyrosine, and the phosphotyrosine, are shown in stick representation. This is the SH2 domain from the Src protein from Rous sarcoma virus. PDB code: 1SPS [Ref. 21]. (E) Schematic of the transduction and selection. ITK-deficient Jurkat cells or BTK-deficient Ramos cells were transduced with a library of viruses containing BTK or chimeras, an IRES, and EGFP. Ramos cells were stimulated with anti-IgM stimulatory antibody at 4 µg / mL. Cells were stained for CD69 and sorted for both EGFP (marking the transduced population) and CD69. An input fraction, taken just before sorting, was used to determine enrichments.

Figure S2

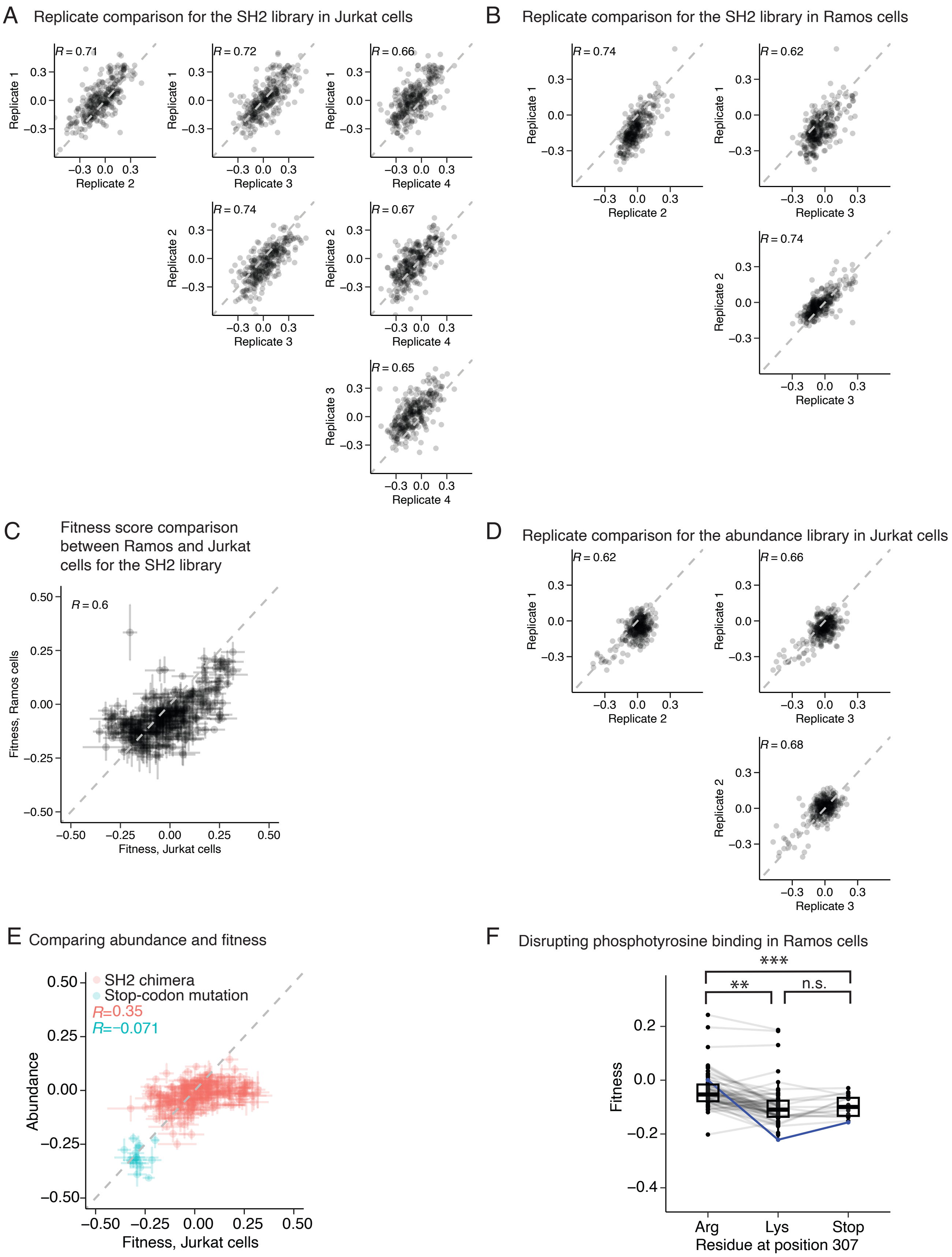
A
Replicate comparison for the SH2 library in Jurkat cells
R = 0.71
R = 0.72
R = 0.66
R = 0.74
R = 0.67
R = 0.65
Replicate 1
Replicate 2
Replicate 3
Replicate 4
−0.3 0.0 0.3
B
Replicate comparison for the SH2 library in Ramos cells
R = 0.74
R = 0.62
R = 0.74
C
Fitness score comparison between Ramos and Jurkat cells for the SH2 library
R = 0.6
Fitness, Ramos cells
Fitness, Jurkat cells
−0.50 −0.25 0.00 0.25 0.50
D
Replicate comparison for the abundance library in Jurkat cells
R = 0.62
R = 0.66
R = 0.68
E
Comparing abundance and fitness
SH2 chimera
Stop-codon mutation
R=0.35
R=−0.071
Abundance
Fitness, Jurkat cells
F
Disrupting phosphotyrosine binding in Ramos cells
***
**
n.s.
Fitness
0.2 0.0 −0.2 −0.4
Arg
Lys
Stop
Residue at position 307

Fig. S2. Reproducibility of fitness and abundance scores. (A) Pairwise comparisons of fitness scores between four biological replicates for the n = 328 SH2 domains using the Jurkat cell assay. The indicated R is the Pearson correlation between the two samples, and the dashed line indicates the y = x diagonal. (B) Pairwise comparisons of fitness scores between three biological replicates for the n = 328 SH2 domains using the Ramos cell assay. The indicated R is the Pearson correlation between the two samples, and the dashed line indicates the y = x diagonal. (C) Comparison between the mean fitness values for each of the n = 328 SH2 domain chimeras in the Jurkat and Ramos assays. The fitness values are the mean of the biological replicates, and the error bars represent the standard error of the mean. (D) Pairwise comparisons of abundance measurements between three biological replicates for the n = 328 SH2 domains using Jurkat cells. The indicated R is the Pearson correlation between the two samples, and the dashed line indicates the y = x diagonal. (E) Comparison between the mean fitness values and abundance measurements for each of the n = 328 SH2 domain chimeras in the Jurkat assay. The values are the mean of the biological replicates, and the error bars represent the standard error of the mean. Sequences containing a stop codon are shown in green and sequences without a stop codon are shown in red. (F) Fitness scores from mutation of Arg 307, the phosphotyrosine-interacting residue (shown in Figure S1D) measured using the Ramos cell assay. SH2 domain sequences derived from the Tec kinases or ancestral-sequence reconstruction were mutated to replace the Arg residue at the equivalent position to 307 in human BTK with either lysine (62 sequences, including BTK) or a stop codon (17 sequences). The blue line is the human BTK SH2 sequence. A box-and-whiskers plot is overlayed with the line (median), box (first and third quartiles) and whiskers (1.5x the interquartile range). Asterisks denote $^{**}P < 0.01$ or $^{***}P < 0.001$ with a one-way ANOVA with Tukey's multiple comparison tests.

Figure S3

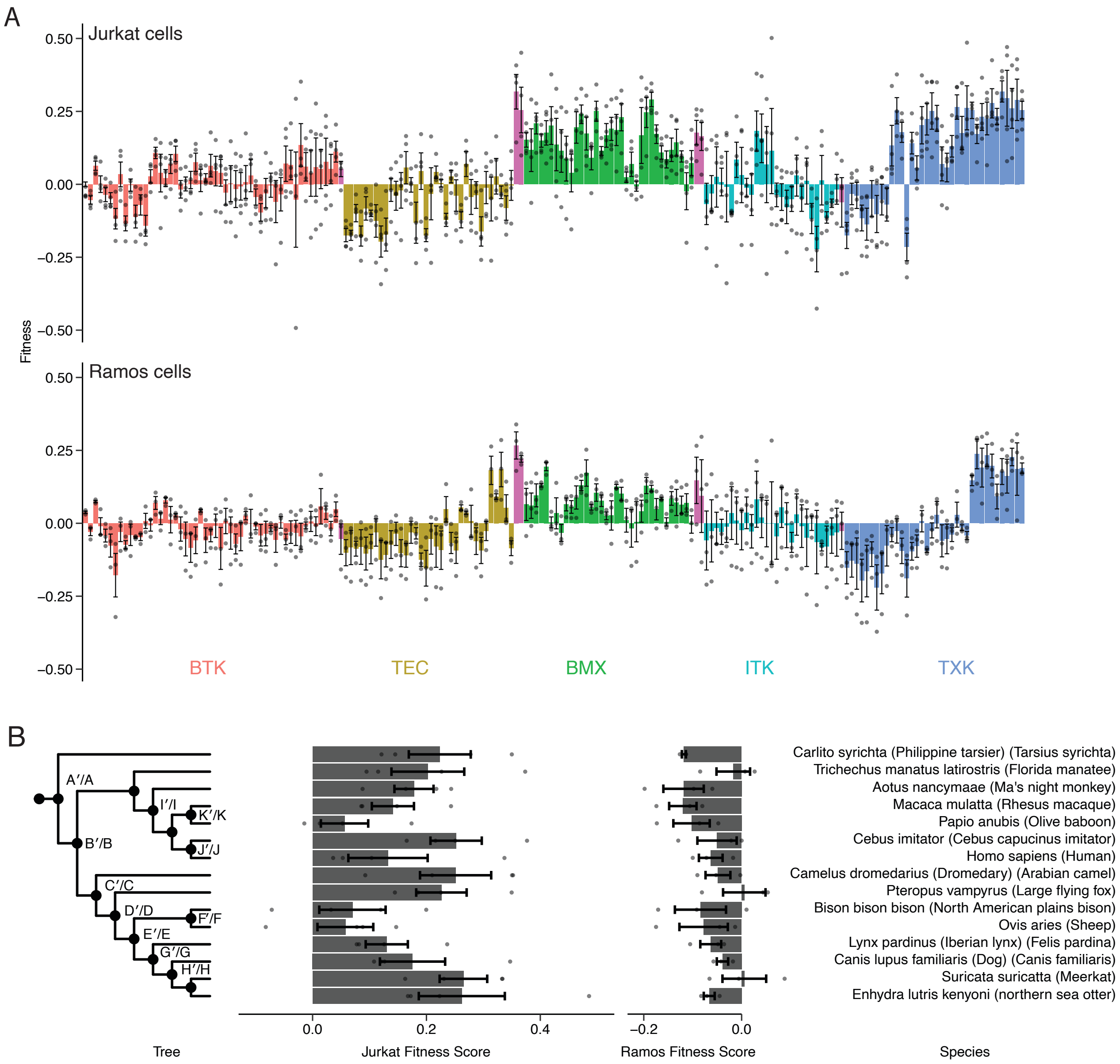
A
Jurkat cells
Ramos cells
Fitness
0.50
0.25
0.00
−0.25
−0.50
BTK
TEC
BMX
ITK
TXK
B
A′/A
B′/B
C′/C
D′/D
E′/E
F′/F
G′/G
H′/H
I′/I
J′/J
K′/K
Tree
Jurkat Fitness Score
Ramos Fitness Score
Species
0.0
0.2
0.4
−0.2
Carlito syrichta (Philippine tarsier) (Tarsius syrichta)
Trichechus manatus latirostris (Florida manatee)
Aotus nancymaae (Ma's night monkey)
Macaca mulatta (Rhesus macaque)
Papio anubis (Olive baboon)
Cebus imitator (Cebus capucinus imitator)
Homo sapiens (Human)
Camelus dromedarius (Dromedary) (Arabian camel)
Pteropus vampyrus (Large flying fox)
Bison bison bison (North American plains bison)
Ovis aries (Sheep)
Lynx pardinus (Iberian lynx) (Felis pardina)
Canis lupus familiaris (Dog) (Canis familiaris)
Suricata suricatta (Meerkat)
Enhydra lutris kenyoni (northern sea otter)

Fig. S3. Comparison between fitness scores in Jurkat and Ramos cells. (A) Comparison of individual fitness scores from SH2 chimeras in the Jurkat and Ramos cells, as in Figure 2. (B) Fitness scores for SH2 chimeras with SH2 domains derived from extant TXK proteins in Jurkat cells and Ramos cells. Species names (Latin and common) are shown at the right.

Figure S4

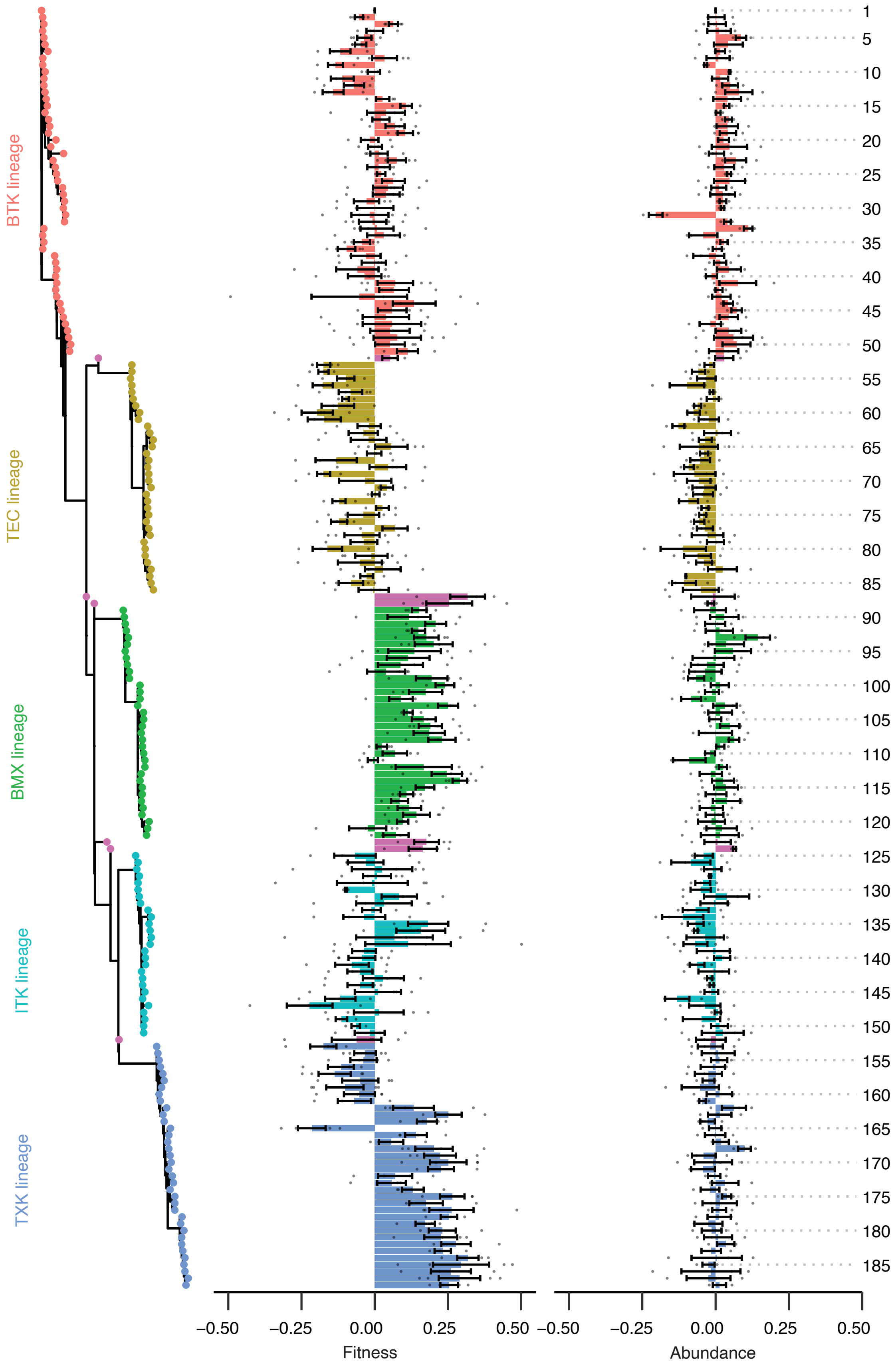

BTK lineage
TEC lineage
BMX lineage
ITK lineage
TXK lineage
Fitness
Abundance
−0.50
−0.25
0.00
0.25
0.50
1
5
10
15
20
25
30
35
40
45
50
55
60
65
70
75
80
85
90
95
100
105
110
115
120
125
130
135
140
145
150
155
160
165
170
175
180
185

Fig. S4. Comparison between the fitness and abundance scores for SH2-domain chimeras. Fitness scores (left) and abundance measurements (right) for the 188 SH2-domain chimeras corresponding to Tec kinases or ancestral SH2 domains. Otherwise as in Figure 2.

Figure S5

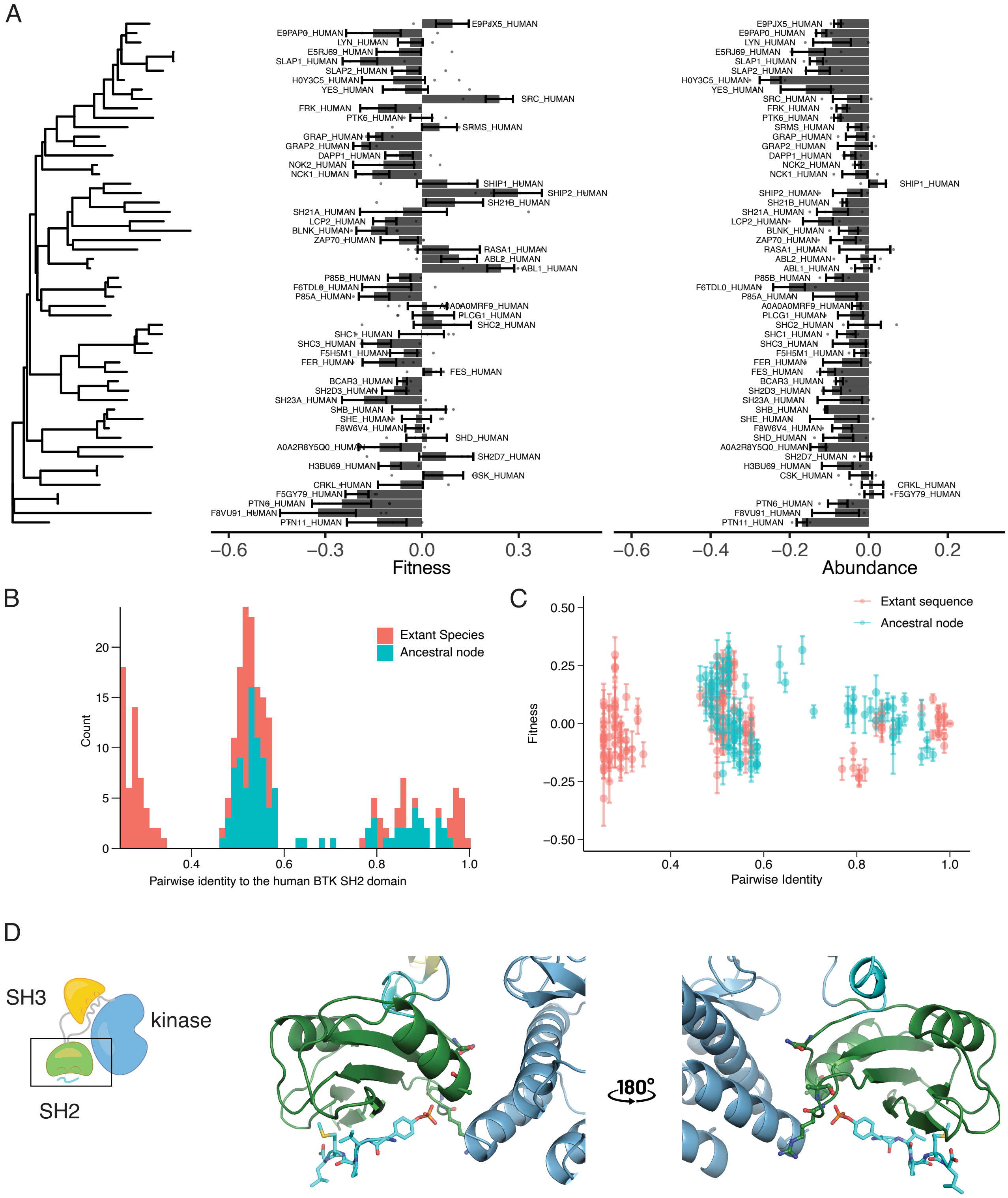
A
Fitness
Abundance
B
Extant Species
Ancestral node
Count
Pairwise identity to the human BTK SH2 domain
C
Extant sequence
Ancestral node
Fitness
Pairwise Identity
D
SH3
kinase
SH2
180°

Fig. S5. Substitution of SH2 domains from distantly related proteins. (A) Fitness scores (left) and abundance measurements (right) for 54 human SH2-domain sequences with between 25% and 40% identity to the human BTK SH2 domain. Sequences are arranged on a phylogenetic tree, with error bars representing the standard error and points representing individual replicate values. (B) A histogram of the pairwise identities of the SH2 domains included in the library, colored by whether the domains are derived from pre-existing proteins or generated using ancestral-sequence reconstruction. Pairwise identities are with respect to the human BTK SH2 domain sequence. Note that the only domains with pairwise identities in between those of the Tec-kinase group (~50%) and the BTK group (~90%) are ancestral, because ancestral-sequence reconstruction allows the generation of sequences with intermediate pairwise identity. The most distant sequences, which consist of SH2 domains from human proteins other than the Tec kinases, were not used for ancestral-sequence reconstruction. (C) Relationship between the fitness score of each SH2 domain and its pairwise identity to the human BTK SH2 domain. (D) Model of the phosphopeptide ligand and the four substitutions in the BMX-A chimera. The phosphopeptide ligand from a crystal structure of an SH2-domain interaction from v-Src [Ref. 21] (PDB: 1SHA) was modeled onto the Alphafold structural model of the BMX-A chimera (Figure 3C). The four substitutions associated with the fitness increase between BMX-A and BMX-H are shown as sticks.

Figure S6

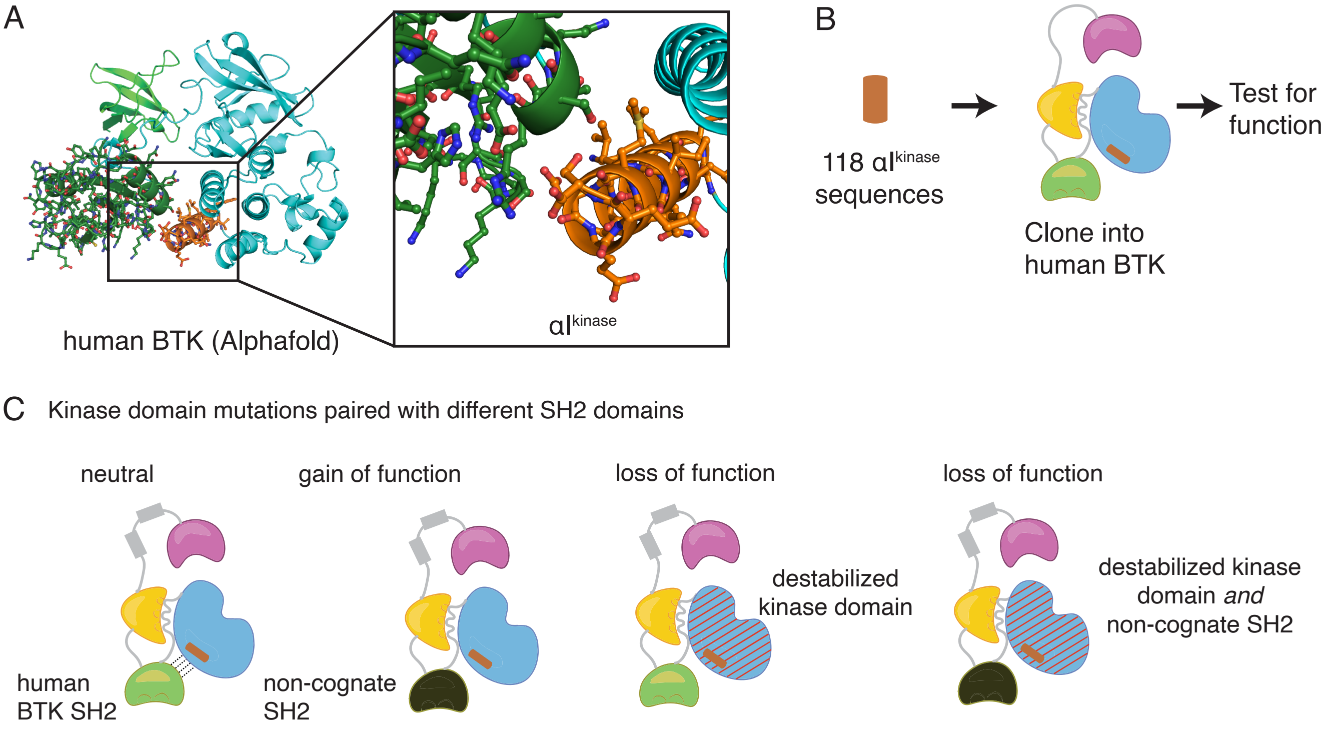

A
human BTK (Alphafold)
αI^{kinase}
B
118 αI^{kinase} sequences
Clone into human BTK
Test for function
C Kinase domain mutations paired with different SH2 domains
neutral
human BTK SH2
gain of function
non-cognate SH2
loss of function
destabilized kinase domain
loss of function
destabilized kinase domain *and* non-cognate SH2

Fig. S6. Substitution of αI^{kinase}. (A) Alphafold [Ref. 25] predicted structure of human BTK, with inset showing αI^{kinase}. The SH2 domain and αI^{kinase} are shown in stick representation. (B) Schematic of the αI^{kinase} swapping experiment. (C) Schematic representation of the effect of combining a destabilizing mutation in the kinase domain with a non-cognate SH2 domain.

Figure S7

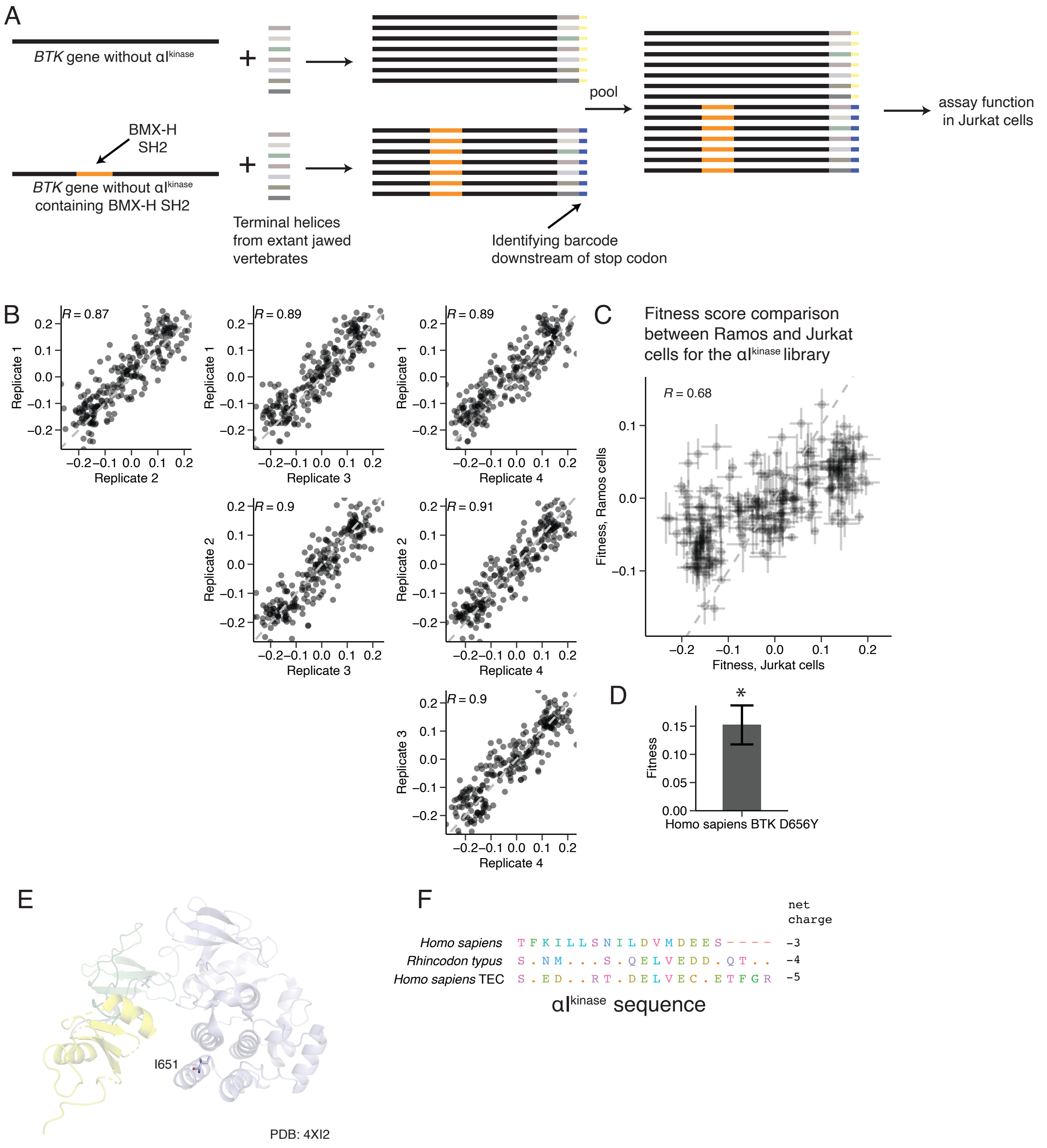
A
BTK gene without αIkinase
BMX-H SH2
BTK gene without αIkinase containing BMX-H SH2
Terminal helices from extant jawed vertebrates
Identifying barcode downstream of stop codon
pool
assay function in Jurkat cells
B
R = 0.87
R = 0.89
R = 0.89
R = 0.9
R = 0.91
R = 0.9
Replicate 1
Replicate 2
Replicate 3
Replicate 4
C
Fitness score comparison between Ramos and Jurkat cells for the αIkinase library
R = 0.68
Fitness, Ramos cells
Fitness, Jurkat cells
D
Fitness
*
Homo sapiens BTK D656Y
E
I651
PDB: 4XI2
F
net charge
Homo sapiens TFKILLSNILDVMDEES---- -3
Rhincodon typus S.NM...S.QELVEDD.QT.. -4
Homo sapiens TEC S.ED..RT.DELVEC.ETFGR -5
αIkinase sequence

Fig. S7. Library construction of the αI^{kinase} sequences in two genetic backgrounds. (A) Schematic of the library cloning for the αI^{kinase} swapping experiment. A library of αI^{kinase} sequences was used to replace the αI^{kinase} sequence in either human BTK or the BMX-H chimera. These two sets of libraries were then barcoded with a 3-nucleotide barcode after the stop codon, and pooled. A single sequencing read was used to determine the identity of αI^{kinase} and the barcode, which indicated whether the SH2 domain was BMX-H or human BTK. (B) Replicate correlations for the fitness scores for the 236 proteins assayed (118 αI^{kinase} variants in two genetic backgrounds). The indicated R is the Pearson correlation between the two samples, and the dashed line indicates the y = x diagonal. (C) Comparison between the mean fitness values in Jurkat and Ramos cells for each of the 118 αI^{kinase} chimeras in both the human BTK SH2 and BMX-H SH2 genetic backgrounds (n = 236 total). Error bars represent standard error of the mean. (D) The D656Y substitution increase fitness in human BTK. An αI^{kinase} sequence containing this single substitution was included in the αI^{kinase} library and assayed in Jurkat cells. Error bars represent standard error of the mean. The asterisk indicates that this substitution is significantly different than 0 with $P < 0.05$ for the n = 4 replicates, t-test. (E) Crystal structure of mouse Btk, highlighting the position of Ile 651. PDB: 4XI2[Ref. 11]. (F) αI^{kinase} sequences of human BTK, *R. typus* BTK, and human TEC kinase. The net charge across the sequence (the number of glutamates or aspartates subtracted from the number of lysines or arginines) is shown at the right.
